# The Evolution of Living Donor Nephrectomy Program at A Hellenic Transplant Center. Laparoscopic vs. Open Donor Nephrectomy: Single-Center Experience

**DOI:** 10.3390/jcm10061195

**Published:** 2021-03-12

**Authors:** Spyridon Vernadakis, Smaragdi Marinaki, Maria Darema, Ioanna Soukouli, Ioannis El. Michelakis, Chrysoula Beletsioti, Georgios Zavvos, Ioannis Bokos, Ioannis N. Boletis

**Affiliations:** 1 Kidney Transplantation Unit, Laiko General Hospital of Athens, 11527 Athina, Greece; gzavvos.md@gmail.com (G.Z.); john.bokos@gmail.com (I.B.); 2 Nephrology and Kidney Transplantation Clinic, Laiko General Hospital of Athens, National and Kapodistrian University of Athens, 15772 Athens, Greece; smaragdimarinaki@yahoo.com (S.M.); mdarema0@gmail.com (M.D.); ioanna_sou@hotmail.com (I.S.); laikneph@laiko.gr (I.N.B.); 3 Department of Hygiene, Epidemiology and Medical Statistics, National and Kapodistrian University of Athens, 11526 Athens, Greece; michgiannis@gmail.com; 4 Department of Internal Medicine, General Hospital of Athens “Korgialenio-Benakio”, 11526 Athens, Greece; 5 Department of Health Economics, School of Medicine, General Hospital of Nikaia “Agios Panteleimon”, National and Kapodistrian University of Athens, 18454 Athens, Greece; chrysa510@gmail.com

**Keywords:** kidney transplantation, living donation, laparoscopic, outcomes, quality of life

## Abstract

Since its introduction in 1995, laparoscopic nephrectomy has emerged as the preferred surgical approach for living donor nephrectomy. Given the ubiquity of the surgical procedure and the need for favorable outcomes, as it is an elective operation on otherwise healthy individuals, it is imperative to ensure appropriate preoperative risk stratification and anticipate intraoperative challenges. The aim of the present study was to compare peri-and postoperative outcomes of living kidney donors (LD), who had undergone laparoscopic nephrectomy (LDN), with a control group of those who had undergone open nephrectomy (ODN). Health-related quality of life (QoL) was also assessed using the validated SF-36 questionnaire. Data from 252 LD from a single transplant center from March 2015 to December 2020 were analyzed retrospectively. In total, 117 donors in the LDN and 135 in the ODN groups were assessed. Demographics, type of transplantation, BMI, duration of surgery, length of hospital stay, peri- and postoperative complications, renal function at discharge and QoL were recorded and compared between the two groups using Stata 13.0 software. There was no difference in baseline characteristics, nor in the prevalence of peri-and postoperative complications, with a total complication rate of 16% (mostly minor, Clavien–Dindo grade II) in both groups, while a different pattern of surgical complications was noticed between them. Duration of surgery was significantly longer in the ODN group (median 240 min vs. 160 min in LDN, *p* < 0.01), warm ischemia time was longer in the LDN group (median 6 min vs.2 min in ODN, *p* < 0.01) and length of hospital stay shorter in the LDN group (median 3 days vs. 7 days in ODN). Conversion rate from laparoscopic to open surgery was 2.5%. There was a drop in estimated glomerular filtration rate (eGFR) at discharge of 36 mL/min in the LDN and 32 mL/min in the ODN groups, respectively (*p* = 0.03). No death, readmission or reoperation were recorded. There was a significant difference in favor of LDN group for each one of the eight items of the questionnaire (SF1–SF8). As for the two summary scores, while the total physical component summary (PCS) score was comparable between the two groups (57.87 in the LDN group and 57.07 in the ODN group), the mental component summary (MCS) score was significantly higher (62.14 vs. 45.22, *p* < 0.001) in the LDN group. This study provides evidence that minimally invasive surgery can be performed safely, with very good short-term outcomes, providing several benefits for the living kidney donor, thereby contributing to expanding the living donor pool, which is essential, especially in countries with deceased-donor organ shortage.

## 1. Introduction

Kidney transplantation (KTx) undoubtedly offers the best therapeutic option for patients with end stage renal disease (ESRD), resulting in substantial improvement not only in quality of life (QoL) but, most importantly, in life expectancy of the recipient compared to renal replacement therapies (RRT) [1]. Compared to deceased-donor (DD) renal transplantation, KTx from living donors (LDs) provides better long-term graft and recipient outcomes; immediate graft function; early access to transplantation, including preemptive KTx; and reduced cost for the health system [2,3,4].

Especially in countries with deceased-donor organ shortage, such as is Greece, expanding the living donor pool is essential, since living KTx comprises almost two-thirds of the total kidney transplant activity of the country.

Living kidney donors represent a unique population, posing a great challenge to both transplant surgeons and nephrologists: they are healthy individuals voluntarily undergoing a surgical procedure of organ removal with a purely altruistic motive, where the recipients benefit. Safety of the surgical procedure and excellent short- and long-term results regarding all health-related outcomes of LD are prerequisites of paramount importance.

Living kidney donation has proven safety in several studies, with low rates of mostly minor complications and minimal mortality [3]. Since its introduction in 1995, laparoscopic donor nephrectomy (LDN) is the gold standard approach for living-donor kidney procurement [4]. When compared to open donor nephrectomy (ODN), LDN is associated with less postoperative pain, faster recovery, shorter length of hospital stay, quicker return to normal daily activities and a better cosmetic result [5]. Consequently, it has been suggested that LDN mitigate some of the disincentives for the live kidney donation, thereby expanding the donor pool for patients with ESRD [6]. Concerning the state-of-the-art indications and techniques/approaches for living donor nephrectomy, the current European Association of Urology (EAU) guidelines on renal transplantation consider LDN as the preferred technique, due to the highly valid evidence confirming its equality to open surgery in terms of patient and graft survival, graft function, urological complications and rejection rates [7]. Moreover, robotic-assisted living donor nephrectomy (RLDN) represents a safe technique and offers a reasonable alternative to conventional laparoscopic surgery in living kidney donation, aiming in maximizing donor safety, minimizing donor discomfort and improving surgeon’s ergonomics and confidence [8,9,10].

The aim of the present study was to investigate perioperative and short-term outcomes of living kidney donors who had undergone laparoscopic nephrectomy (LDN), compared to a control group of patients after open donor nephrectomy (ODN) at our institution.

## 2. Materials and Methods

All consecutive living kidney donors (*n* = 252) from the Renal Transplant Unit of Laiko General Hospital of Athens from March 2015 to December 2020 were analyzed retrospectively. From its introduction in October 2018 in our center, LDN was the standard nephrectomy procedure in all living donors, with very few exceptions, due to logistic reasons. Data from all (*n* = 117) living donors who had undergone laparoscopic nephrectomy were collected. All consecutive living donors who had undergone open nephrectomy (*n* = 135) from that time backward until March 2015 served as control group. The study was approved by the local ethics committee.

All donors had successfully completed extensive pre- and perioperative work-up, including computed tomographic angiography scan to define their vascular anatomy, in order to ensure that it was amenable for transplantation. If we found a large size discrepancy between the two kidneys, we obtained a split-function renogram (DMSA) to individually assess the functional status of each kidney. If both kidneys were suitable for transplantation, for all donor nephrectomies (whether open or laparoscopic), if there was a single artery, our practice was to prefer the left kidney. If there were two renal arteries or more on the left kidney and a single artery on the right kidney, we would take the right one. If there were multiple arteries bilaterally, we removed the left kidney. If there was an incidental minor abnormality (e.g., a simple cyst), we removed the kidney with the abnormality, leaving the living donor with the normal kidney.

Data collected on living donors included transplantation characteristics, i.e., preemptive or ABO-incompatible transplantation; donor and recipients demographics; donors’ comorbidities and BMI; duration of operation; warm ischemic time (WIT); length of hospital stay; any intra- and postoperative complications, according to the Clavien–Dindo [11] classification (CDC) of surgical complication; and any postoperative short-term (≤30 days) complications (defined as untoward events within the postoperative period that affected their recovery, prolonged their hospital stay or required technical deviations during the surgical procedure). Renal function of donors at surgery and at discharge was also analyzed.

Furthermore, health-related quality of life (HRQL) was assessed using the standardized SF-36 questionnaire in both donor groups. The Short Form-36 (SF-36, Greek version 2.0) questionnaire is a generic measure of perceived health status. The SF-36 includes 36 items, which can be aggregated into eight scales: physical functioning (PF), physical role (RP), bodily pain (BP), general health (GH), vitality (VT), social functioning (SF), emotional role (RE) and mental health (MH). These eight scales yield two summary measures: physical component summary score (PCS) and mental component summary score (MCS) [11]. We compared the scores for each item and for the two summary scores between the two groups. Both LD groups were contacted personally via telephone interview by a person not directly associated with the transplant team. Median time from surgery to evaluation was 42 ± 13 (range 5–71) months in the open nephrectomy and 11 ± 7 (range 1–26) months in the laparoscopic nephrectomy groups, respectively. The response rate was high: questionnaires were completed by 122 out of 132 (92.4%) of donors in the open nephrectomy and by 119 out of 120 (99 %) of donors in the laparoscopic groups, respectively.

### 2.1. Statistical Analysis

Continuous variables were expressed as mean and standard deviation, and categorical variables as frequency and percentage (%). To investigate the differences between clinical variables in patients who underwent open or laparoscopic nephrectomy, the t-test and Mann–Whitney U test for independent samples for continuous variables and the chi-square test for categorical variables were applied. Data were analyzed using Stata 13.0 software (Stata Corporation, College Station, TX, USA). Significance was set at α = 0.05, and all tests proceeded as two-tailed.

As for the SF-36 questionnaires, a scoring algorithm, recommended by the developers, codes, sums and transforms the responses per domain into a scale from 0 to 100, with higher scores indicating better health status. Specifically, after the data entry, the item recoding procedure takes place by changing out-of-range values to missing. Subsequently, recoding values for 10 items is conducted to ensure that a high score indicates better health status and to satisfy the equal interval assumption, and missing item responses are recoded with mean substitution when the respondent has answered at least half of the items in a multi-item scale. Finally, raw scale scores are computed and transformed to a 0–100 scale [12].

Additionally, summary components are computed through a standardized three-step process that adds together the scores from all domains with differing weightings, based on the principal component analysis method. First, the eight dimensions are standardized using a z-score transformation; second, z-scores are multiplied with the respective factor score coefficient and summed in order to compute aggregate scores; and finally, the obtained scores are standardized using a T-score transformation (mean 50 and SD 10) [11]. The SF-36 has been translated, validated and normed for the Greek population by Pappa et al. [13]. The analysis was performed with IBM Statistical Package for the Social Sciences for Windows version 25.0 (SPSS, IBM Corp., Armonk, NY, USA).

### 2.2. Surgical Techniques

All surgical procedures were performed in the Renal Transplant Unit of “LAIKO” General Hospital of Athens. All eligible donors who were considered for kidney donation were informed about the surgical approach. Standard preoperative screening of donors included examination by a nephrologist, a transplant surgeon and an anesthetist. Three transplant surgeons performed all the surgical procedures in both donor groups, whereas only one performed all LDN. Regarding the selection of the surgical procedure of the kidney living donors, surgeon experience and donor factors, such as BMI and kidney anatomy, were taken into account. During the period prior to October 2018, the ODN was the only one surgical option that our center has offered. This kind of donor nephrectomy was carried out by three different surgeons with appropriate experience; two of them had no experience with laparoscopic donor nephrectomies. Since October 2018, a well-codified minimally invasive LDN surgical program has been established; this was followed by standardization of the surgical procedure and development of a well-defined surgical team, including a surgeon highly skilled in laparoscopic and robotic surgery and transplantation surgery, who was previously trained in other transplant centers and who has performed all LDNs.

#### 2.2.1. Donor

(1) LDN

**Figure 1:**
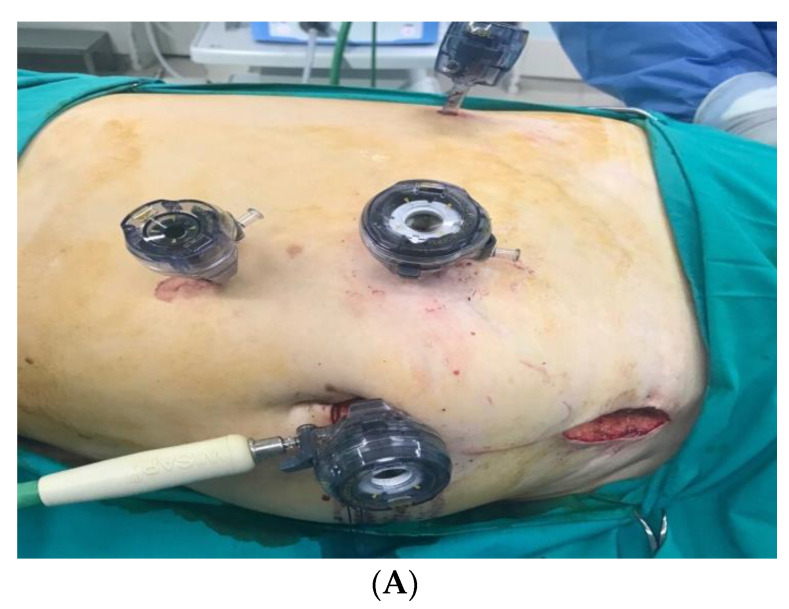

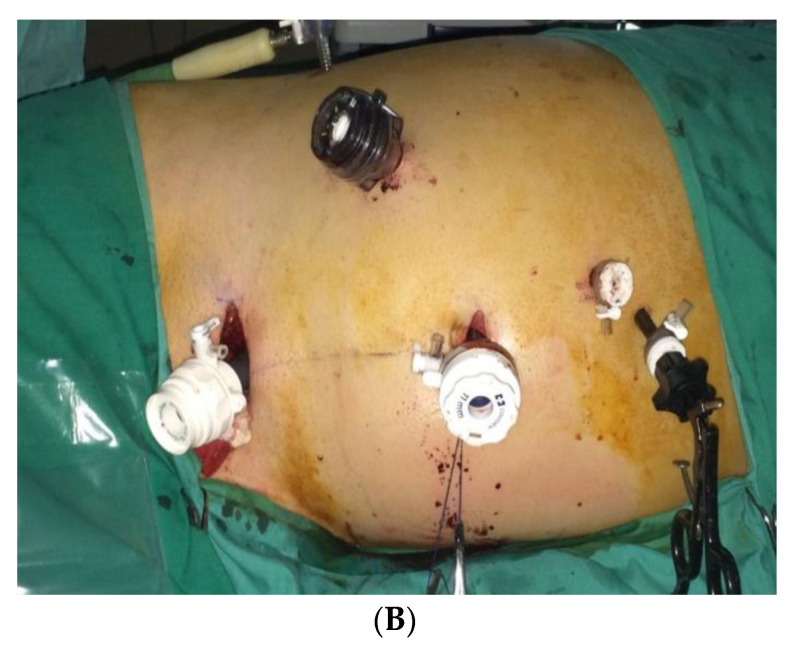
Port placement for laparoscopic donor nephrectomy (LDN). (**A**) Overview of the port placement for left-sided LDN. Four trocars. (**B**) Overview of the port placement for right-sided LDN. Five trocars.

**Figure 2:**
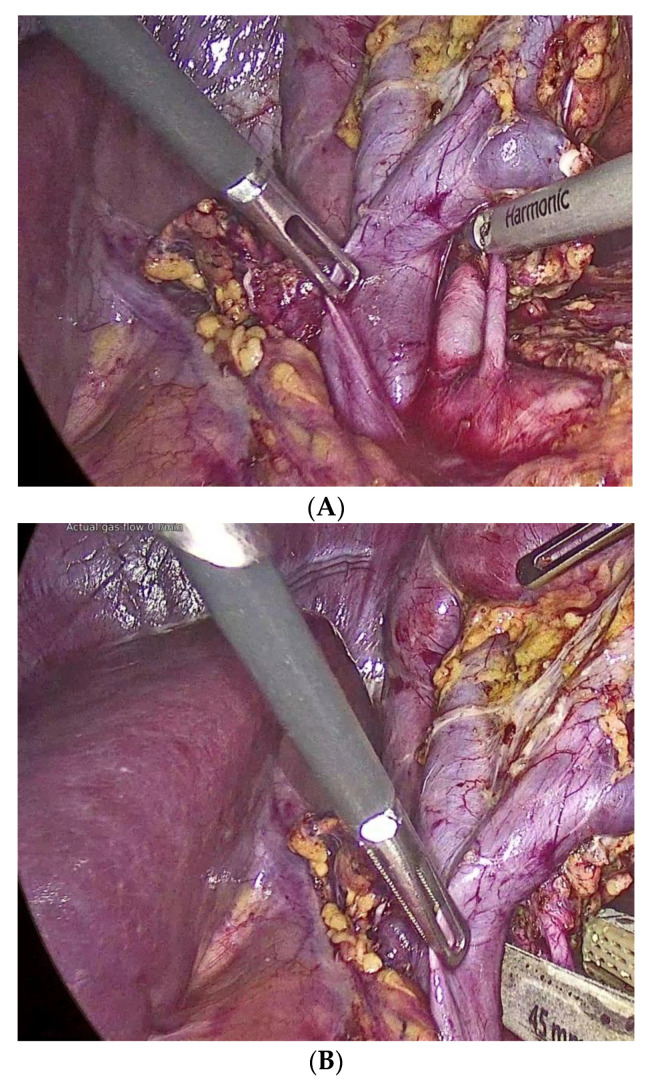

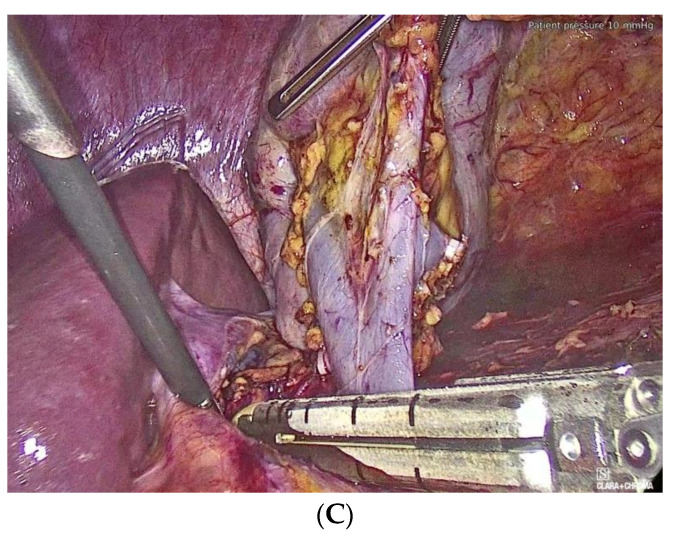
Port placement for laparoscopic donor nephrectomy (LDN). (**A**) Dissection of the renal arteries. (**B**) Control of the renal arteries with the vascular Endo-GIA. (**C**) Control of the renal vein with the vascular Endo-GIA.

The procedure was performed with the donor in lateral decubitus position. A 10 mm was introduced under direct vision. The abdomen was insufflated to 12 cm H_2_O carbon dioxide pressure. A 30° video endoscope was introduced. Three to four trocars were introduced in the peritoneal cavity under direct vision. The colon was mobilized and displaced medially. After opening the Gerota’s fascia and division of the perirenal fat, the ureter and vascular structures were dissected. A 5–7 cm Pfannenstiel (suprapubic transverse) incision was made as extraction site. The ureter was clipped and divided, and the renal vessels were stapled. The kidney was recovered with an endobag (Figure 1 and Figure 2).

(2) ODN (Flank incision)

**Figure 3:**
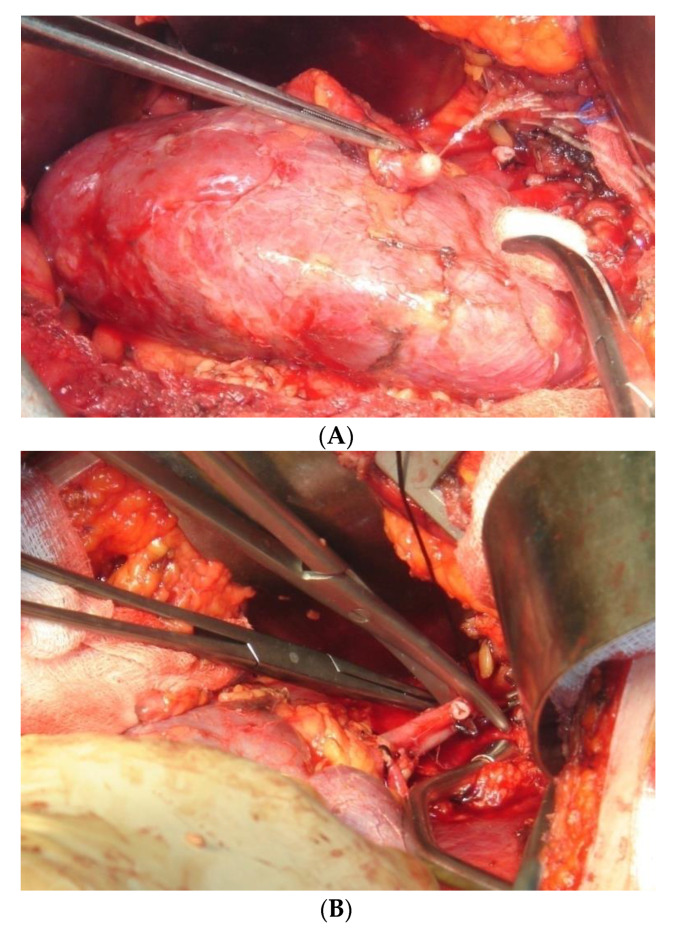
Port placement for laparoscopic donor nephrectomy (LDN). (**A**) Ligation of the ureter. (**B**) Ligation of the renal vessels.

With the donor placed in a lateral decubitus position, lumbotomy was performed with a horizontal 20–30 cm skin incision anterior to the 11th rib. A mechanical retractor was used. The muscles of the abdominal wall were split. Gerota’s fascia were opened on the lateral side of the kidney. After dissection of the kidney and its structures, the ureter, renal artery and vein were subsequently divided, and the kidney was extracted. The fascias of the abdominal muscles were closed, and the skin was clipped or sutured intracutaneously (Figure 3).

#### 2.2.2. Recipient

Preperitoneal placement of the renal graft in the iliac fossa was applied as the standard operation technique.

## 3. Results

For this study, the following data were collected: age, sex, BMI, relation to recipient, operative time (OT), side of operation, perioperative bleeding, prior abdominal surgery, number of renal arteries, other anatomical variants, warm ischemic time (WIT), intraoperative complications, conversion to open surgery, reason for conversion, postoperative complications and length of hospital stay. The OT was from skin to skin. WIT was recorded and documented in the operating theater by a coordinator or nursing staff. The time recorded was from when the artery was clamped and stapled until the kidney was immersed in ice sludge on the back table and flushing of the artery with cold perfusion solution began. Postoperative hospital stay is from the first postoperative day until discharge.

### 3.1. Donor Characteristics

Transplantation and donor characteristics in both ODN and LDN groups are listed in Table 1. There was no difference in baseline donor characteristics between the two groups. Our donors were predominantly female (71% in the open and 74% in the laparoscopic nephrectomy group), aged 58 ± 9 years, with a mean preoperative BMI at 26.5 and 27 kg/m^2^ and mean preoperative estimated glomerular filtration rate (eGFR) of 93 and 96 mL/min, respectively. Left kidney procurement was the preferred surgical approach: the left kidney was removed in 87.4% of donors in the ODN group and in 86.3% in the LDN group. The only significant difference between the two groups was the frequency of past abdominal surgery: a substantially higher proportion of donors in the LDNgroup (39%) had undergone abdominal surgery prior to nephrectomy, compared to 18.2% in the ODN group (*p* < 0.001).

Age distribution was similar between groups. As shown in Table 2, a substantial proportion of donors in both groups were above 60 years, 47% in the ODN group and 39% in the LDN group, while the percentages of those aged over 65 years were 27% in the ODN and 26% in the LDN groups, respectively.

Mean donor BMI did not differ between groups. However, obese donors (BMI > 30 kg/m^2^) were more prevalent (25% vs. 12%, *p* =0.08) in the LDN group.

Preemptive kidney transplantation (KTx) was performed in 18.5% of cases in the ODN group and in 13.5% in the LDN group and ABO-incompatible KTx in 7.4% and 29%, respectively.

Comparison of major outcomes between groups is depicted in Table 2. Donors’ eGFR at discharge dropped from a baseline preoperative value of 93 mL/min (IQR 86–100 mL/min) to 60 mL/min (IQR 52–71 mL/min) in the ODN group and from a median of 95.5 ml/min (IQR 83.5–101 mL/min) to 59 mL/min (IQR 48–65 mL/min) in the LDN group. This corresponds to a ΔeGFR of 32 mL/min (ODN) and 36 mL/min (LDN) groups, respectively (*p* =0.03).

### 3.2. Intraoperative Parameters

Intraoperative parameters by surgical technique are reported in Table 3.

The duration of surgery (operative time in min) was counted from skin to skin. In our study, ODN required an operative time that was, on average, 80 min longer than LDN (*p* < 0.01).

LDN was associated with significantly longer warm ischemic time (WIT) (2 min–6 min; *p* < 0.01).

LDN and ODN cohorts are comparable in the estimated blood loss and postoperative transfusion requirement, where only one patient in the LDN group lost about 50 mL of blood intraoperatively, while the median blood loss in the ODN group was 20 mL. Only two patients in the ODN group required postoperative transfusion.

The prevalence of intra- or postoperative complications showed no statistical difference between the two groups, ODN vs. LDN (16.3% vs. 15.4%) and is reported in Table 3.

The 12 intraoperative complications regarding the LDN cohort were CDC II in severity and required no surgical or other intervention (e.g., three small spleen lacerations, two lacerations of the renal graft capsule from the endobag, one hematoma of latissimusdorsi muscle, two small bleedings from an accessory suprarenal vein, three cases with bleeding from a small lumbar vein branch and one case of bleeding of the renal artery stump due to atherosclerosis-related misfiring of the endostapler).

The conversion to open rate within the LDN group was 2.56% (3 cases in 117 surgical procedures). Of note, there were no intraoperative or immediately postoperative living donor deaths in our study.

### 3.3. Postoperative Outcomes

Postoperative outcomes for our cohort are listed in Table 3.

LDN was associated with a significant reduction in the average length of hospital stay (ODN 7 days; LDN 3 days (*p* < 0.01)).

Rates of relevant postoperative complications were comparable in the different access groups, ODN vs. LDN (16.3% vs. 15.4%). In addition, the severity of complications between the examined groups was comparable (CDC II: ODN 9.4%; LDN 15.6% or CDC III: ODN 6.7%; LDN 0%) and are reported in Table 3. While significant wound infection and postoperative pain requiring prolonged analgesics and hernia formation or abdominal wall relaxation were, more often, complications of the ODN group, the LDN group experienced not-infected wound seromas and chylousascites that were treated conservatively with more frequent changing of wound dressings and adapted oral diet with low lipid, high medium-chain triglycerides alimentation.

Of interest, no readmissions have been documented in both access groups.

### 3.4. Quality of Life of the Donors

The median SF-36 scores in both groups are presented in Table 4.

There was a significant difference in favor of the LDN group for each one of the eight items of the questionnaire (SF1–SF8). As for the two summary scores, while the total physical component summary (PCS) score was comparable between the two groups (57.87 in the LDN group and 57.07 in the ODN group), the mental component summary (MCS) score was significantly higher (62.14 vs. 45.22, *p* < 0.001) in the LN group. Of note, donors with laparoscopic nephrectomy had undergone surgery more recently than controls: time since nephrectomy in this group was 11 ± 7 months, compared to 42 ± 13 months in the control group of donors with open nephrectomy.

## 4. Discussion

First described over 25 years ago, LDN is the standard operation for retrieval of kidneys for living donation. Given the ubiquity of the surgical procedure and the need for favorable outcomes, as it is an elective operation on otherwise healthy individuals, it is imperative to ensure appropriate preoperative risk stratification and anticipate intraoperative challenges [4,14].

The surgical practice has evolved from the open lumbotomy, through mini-incision donor nephrectomy, to minimally invasive laparoscopic techniques. There are different minimally invasive techniques, including standard pure laparoscopic, hand-assisted laparoscopic, hand-assisted retroperitoneoscopic, pure retroperitoneoscopic, and robotic-assisted live donor nephrectomy [15,16]. Of note, while LDN has become a common practice in most transplant centers around the world due to the benefits that it provides for the donor, the use of robotic-assisted LDN might further improve the outcomes of the procedure, providing distinct benefits, not only for the donor, but also for the surgeon, based on the enhanced dexterity, ergonomic and subsequent confidence of the surgeon that allow for a more standardized and potentially easier procedure, compared to standard laparoscopy, especially in difficult donors with high BMI or complex vascular anatomy [8,9,10].

Several studies, including a meta-analysis [17], have investigated perioperative outcomes between ODN and LDN techniques [18,19]. Recently, a meta-analysis focusing on long-term outcomes of donors with laparoscopic versus ODN has been published [20]. Our study investigated peri- and postoperative as well as health-related QoL parameters in LKD after ODN and LDN. A total of 252 living donors (LDs), 117 with LDN and 135 with ODN from a single, academic, tertiary institution from 2015 to 2020, were analyzed retrospectively.

At our institution, conventional ODN was abandoned in October 2018 in favor of the transperitoneal laparoscopic donor nephrectomy (TLDN) technique. The rationale for this choice was based on morbidity issues and concerns with ODN, which represents a major surgical trauma that causes postoperative pain, discomfort, and more long-term complications to healthy volunteers who have no direct therapeutic benefit of the procedure [5,6]. Since the implementation of LDN in 2018, almost all LKD in our center undergo LDN. Only two LDs have undergone ODN since then, because of internal logistic reasons. Our control group consisted of consecutive LDs with open nephrectomy from 2018, backward.

LDN is a difficult procedure that requires extensive experience in minimally invasive laparoscopy and is surely a more demanding and challenging procedure than ODN. Exact effects, risks and limitations of learning curves in LDN are difficult to estimate. Descriptions vary, from 20% major complications during the first 50 cases and 6% during cases 200–250 to 30% major complications during the first 30 LDN cases with no complications during the following 50 cases [4,5,21,22,23]. In our center, LDN is carried out by only one general and transplant surgeon, who has the expertise to perform laparoscopic kidney donation after sufficient training and experience in advanced laparoscopic surgery. LDN represents surgeon’s personal preference, while experience with other minimally invasive surgical techniques for live donor nephrectomy exists.

In our experience, we observed a relevant procedure-related learning curve, and some of our complications and results (prolonged WIT, small organ lacerations, bleeding from venous branches, two conversions at the beginning of the series) might have been related to such a learning curve. Regarding the selection of the donor nephrectomy surgical procedure, surgeon experience and donor factors, such as BMI and kidney anatomy, were taken into account. In general, ODN was the only option offered during the period prior to October 2018. On the other hand, LDN was the first option used at our institution since October 2018. In the early phase of the learning curve in cases with BMI >30, ODN was considered. In our experience, taking into account surgeons’ prior training and experience in advanced laparoscopic and robotic-assisted procedures, it was relatively safe to develop and implement a new minimally invasive program for LDN, while, according to our results, we estimate the mean number of consecutive cases necessary to reach proficiency in LDN to be between 15–20.

The two groups had similar baseline characteristics. Almost two-thirds of donors in both groups were over 60 years old. Remarkably, obesity, which poses technical difficulties, was more prevalent in the LDN group.

As safety of the donor is the primary consideration in living donor surgery, all major peri- and postoperative outcomes were thoroughly investigated and compared between the two groups.

Perioperative mortality rate, though existing, is minimal and has been reported at 0.03 % [20]. In our cohort, neither donor died as a result of peri- or postoperative complications; moreover, there was no reoperation or readmission during the first 30 days due to surgical complications. The overall perioperative complication rate in our study was similar between groups, at 15.4% in the LDN and 16% in the ODN groups, respectively, similar to complication rates that have been reported by others [6,16,17,24,25]. Overall complication rates in smaller studies range from 6–18%. This, however, depends greatly on the reporting rates and the definitions used [19,26].

### 4.1. Conversion Rate

In a recent European Update survey, the median conversion rate was 2% (range, 1–7%). The median case volume in centers that converted one or more procedures was significantly higher than centers that did not [24,27].Our study population included three patients (3/117), 2.56%, in whom the laparoscopic procedure was converted to an open procedure. The reason for the conversion was not bleeding, which represents the most common conversion reason [6,16,21,25], but enormous difficulties in dissection of the renal vessels due to the amount of the perinephric fat in otherwise not extreme obese male donors (BMI was 28, 29 and 32, respectively). In regards to operative difficulty, we noticed a not-statistically significant correlation to donor male gender and thickness and consistency of perinephric fat.

Obesity has been associated with an increased risk for perioperative complications. Notably, in our cohort, though obesity was more prevalent in the LN group (*p* < 0.008), there was no difference in complication rates. As obesity becomes increasingly common, more potential kidney donors are likely to be obese as defined by BMI, with 63.6% of donors classified as overweight or obese in a review of 2015 [28,29].Though BMI is correlated with both operative difficulty and postoperative donor and recipients outcomes, it is a frequently criticized measure, and inconsistent results have been reported [30]. It may be incorrectly elevated or depressed in certain populations and may not adequately account for specific anatomy [31]. For example, perinephric fat has been shown to be thicker and harder in males than in females, regardless of BMI [32]. Additionally, it is increasingly understood that measures of visceral fat are superior to BMI for delineating those patients with more severe metabolic syndromes [33].A recent paper by Ahmandi et al. recommended not basing donor eligibility solely on BMI values [34].

Our transplant center currently excludes patients with a BMI of 35 kg/m^2^ or higher, while our transplant program’s low acceptance threshold individualizes the decision to approve obese donor candidates with BMI >35 kg/m^2^ based on demographics, health profile and donor habitus characteristics.

Even if these variables were not significant, we strongly believe that male gender and perinephric fat thickness are strongly correlated with operative time and conversion rate, while perinephric fat is not as well-correlated with BMI. This confirms the importance of perinephric fat over BMI when planning donor nephrectomies. As BMI of the general population increases, it may be important to find more specific measures of operative difficulty when considering a weight or BMI cutoff.

Virtually all donors eligible for an open surgical procedure may also undergo the laparoscopic operation. Various earlier contraindications to LDN, such as right donor kidney, multiple vessels, anomalous vasculature and obesity have been overcome with increasing experience [16,22,24,25,27]. The contraindications to kidney donation are similar in both open and laparoscopic procedures and focus on maintaining acceptable long term renal function in the donor. Although a history of multiple abdominal surgeries in the donor may be considered a relative contraindication, a pure laparoscopic approach in a donor with previous transperitoneal surgery, and vice-versa, may still be safely attempted. In our study, similar to the experience of others, a significant difference between the two groups was the frequency of past abdominal surgery: a substantially higher proportion of donors in the LDN group (39%) had undergone abdominal surgery prior to nephrectomy, compared to 18.2% in the ODN group (*p* < 0.001). Of interest, we combined other surgical procedures like cholecystectomy, umbilical hernia repair and incisional hernia repair in six cases, even in donors with previous major abdominal surgeries.

### 4.2. Operative Time

In most reported experiences, the operating time at the beginning of the LDN learning curve was generally longer than for ODN. In a meta-analysis of published reports regarding the current status of LDN, the operating time was significantly longer in LDN series compared with open donor nephrectomy series [17]. However, with increasing experience, it decreased and tended to plateau after about 25–30 cases [16,18,24]. Our median operative time for transperitoneal LDN is 160 min, and sometimes we have finished the operation in 120 min. In our current study, ODN required an operative time that was, on average, 80 min longer than LDN (*p* < 0.01). The reason for this finding is that, during the most of the ODN cases, the surgeon was waiting for the recipient, who was operated on simultaneously, to be prepared for transplantation in order to remove the kidney graft, having a pause of at least 60 min.

### 4.3. Blood Loss

LDN and ODN are comparable in the estimated blood loss and postoperative transfusion requirement [17,18]. In fact, most studies reported lesser blood loss in the laparoscopic group compared to the open group [15,28]. We have found no significant difference in blood loss and transfusion need as seen in many other series.

### 4.4. Warm Ischemic Time

WIT represents a major concern, as it has always been slightly longer in the laparoscopic group when compared with ODN, due to the longer extraction time. It has been thought that any increase in this would translate into a poor graft function. This notion has been disproved by various studies, suggesting no bearing of this small difference on the recipient outcome [16,25,27]. In our study, LDN was associated with significantly longer WIT, compared to ODN (6 min–2 min; *p* < 0.01). This fact seems to be associated with some problems in the use of the extraction endobag and the dissection of the kidney upper pole, especially in obese donors, during the beginning of the learning curve, which required prolonged extraction time.

The WIT can be reduced with increasing experience, as shown in different studies [16,23,35]. Prolonged WIT did not appear to have an effect on early graft function or the rate of decline in serum creatinine during the first 3 months post-transplantation.

We evaluated the impact of warm ischemia time in LDN. It correlated with the incidence of delayed graft function. In two patients, where it took 25 min and 15 min to retrieve the renal grafts, both the kidneys worked well and had diuresis in the immediate postoperative period.

### 4.5. Renal Function of Living Donors at Discharge

In our current study, we noted minor but statistically significant differences in the creatinine level and eGFR at discharge between the LDN and ODN cohorts at baseline. Regarding renal function, there was a drop in eGFR from 93 to 60 mL/min in the ON and from 95.5 to 59 mL/min in the LN group, which corresponds to a ΔeGFR of 36 and 32 mL/min, respectively (*p* < 0.03). Though marginal, the difference was in favor of the ON group. The reported decline in eGFR in both our donor groups is in the expected range, since, at 6 weeks postdonation, the reported declineis 33%in young donors (18–30 years old) and 35–37% in older donors. After that, an average increase in eGFR +0.35 mL/min annually, is expected [36]. These differences could be partly explained by the number of more “complex” donors within the LDN group (older age, obesity, previous abdominal surgery, comorbidities). Factors associated with a higher eGFR decline, such as low predonation eGFR, older age and increased BMI were all prevalent in our cohort. These differences tend to equalize over time; moreover, long-term creatinine postdonation seems to be unaffected by the surgical technique used [11].

### 4.6. Hospital Stay and Convalescence

LDN has been demonstrated to reduce the postoperative analgesic requirement, hospital stay and the length of convalescence, as compared to ODN [5]. Comparing the two different surgical techniques, the hospital stay, resumption of oral intake and the time to return to home have been found in our study to be significantly favorable to the LDN group. LDN was associated with a significant reduction in the average hospital length of stay (ODN 7 days; LDN 3 days (*p* < 0.01)) which, except for faster recovery, is particularly important for the safety of the donors, especially in the times of the COVID-19 pandemic.

### 4.7. Postoperative Pain

Reduced postoperative pain and recuperative time, the major advantages of LDN, have been demonstrated in several studies [6,16,22]. Ratner et al. [4] concluded that the amount of parenteral analgesia given to donors after LDN was significantly lower than after open donor nephrectomy (*p*< 0.05). Similar to the experience of others, we observed remarkably higher incidence of postsurgical pain in ODN, which could be explained by the refinement in surgical techniques with less intraoperative nerve injury in the LDN. In accordance, ODN is a more invasive procedure compared to LDN, because of the use of a muscle-splitting flank incision instead of an intermuscular Pfannenstiel incision [22,25].

### 4.8. Quality of Life Parameters

Several series have addressed donor quality of life following LDN and ODN. It has been shown that LDN resulted in a shorter time until patients were able to return to normal physical activity and work [16,25].

As health-related QoL is a major concern in living kidney donors, several studies have investigated this issue using standardized quality of life scales, including the SF-36 questionnaire. Most studies have reported excellent outcomes in LDs, with LDs scoring even better in many items of the questionnaire, compared to the general population, which can be explained by psychological benefits, such as enhanced self-esteem as a result of donation [24,37,38,39,40]. In a multicenter cohort study comparing 203 living donors with 104 healthy nondonors, both groups scored similarly for all items of the SF-36 [41]. Few studies have directly compared laparoscopic and ODN in terms of QoL with controversial results [42,43]. Some have found significantly higher scores in some but not all items of the questionnaires, while others reported no differences in scoring in either item of the SF-36 survey. In a recent meta-analysis investigating long-term outcomes of laparoscopic vs. ODN, health-related QoL was assessed only in two studies. There was no difference between groups in either item of the SF-36 [20].

In our study, all donors in both groups scored highly in each one of the separate eight items of the SF-36 questionnaire (Table 4). There was a significant difference in all eight items, as well as in the summary MCS score in favor of the LDN group. There are many explanations for this finding: minimally invasive surgery, shorter hospital stay, faster recovery postoperatively and, most importantly, reduced surgical pain, are all factors positively influencing postdonation QoL. Especially for the SF-36 item (BP, bodily pain), it is expected that donors in the LN group score higher, as has also been reported by others [44].

Donors in the LN group had shorter time from donation until completion of the survey. However, time since donation does not seem to influence the SF-36 scores [38,40,45]. The impact of donor age on self-reported QoL is also controversial across studies. Some have reported worse scoring in younger donors (aged 31–40 years), especially for the item SF-3 (bodily pain) [40], while others have reported worse scoring in donors older than 50 years [45]. We did not perform a subgroup analysis; however, considering the high scores in all SF-36 items in all LDs of our cohort and given the fact that 27% of our LDs were above 65 years old, we could demonstrate in our study that older age did not negatively influence QoL after donation.

### 4.9. Expansion of the Living Donor Pool

Long-term follow-up data, especially for “extended criteria” or “complex” living donors (e.g., donors with obesity, older age, hypertension, vascular multiplicity) are lacking, as these donors have been increasingly accepted, mainly during the last decade. It has been shown that donors with overweight/obesity and donors with vascular multiplicity have good short-term results [46].

Previous studies demonstrated that older living donors have excellent outcomes [27]. More than half of the centers have no age limit for the donors; however, geographical differences exist. In the USA, there seems to be a stricter policy regarding donor age. On the other hand, British guidelines state that older age represents, not an absolute contra-indication for donation, but that the medical work up of these donors should be particularly rigorous to ensure suitability [34,46].

LDN minimizes the drawbacks of ODN and makes the prospects of live donor nephrectomy more appealing to prospective donors by reducing postoperative pain, shortening convalescence and improving the cosmetic outcome of donor nephrectomy. LDN has shown the potential to increase the number of living kidney donations by removing some of the disincentives inherent to kidney donation itself [14,24,47]. It is evident that the annual number of available deceased donors will not resolve the growing organ shortage and the prolonged waiting time for transplantation. In Europe, patients wait on average 3–5 years for a deceased donor kidney [47], while in Greece, the average waiting time goes up to 7–9 years (data source: Greek Transplant Organization; 2020). Greece is currently facing very low organ donation rates, which is influenced by a variety of factors, including medical, cultural, legal, ethical and socioeconomic aspects, which has resulted in rising morbidity and mortality for patients on the waiting list [48].

To enlarge the donor pool, the use of living donors in Europe has increased. Many centers have, therefore, placed greater emphasis on living kidney donation to meet the growing demand for kidney transplantation in patients with ESRD [14,47,49].

Our transplant center moved toward the same direction. It is the biggest in Greece, with the most organized living donor program. We strongly believe that the initiation of the LDN in our center, which is the only one at the moment in the country that regularly performs minimally invasive donor nephrectomies (data source: Greek Transplant Organization; 2020), has given impetus to the expanding of our live donor kidney transplant program. Over the past three years we have performed 181 renal transplantations from LDs, with 48 of them having been performed in 2018, 58 in 2019 and, of interest, 75 in 2020, despite having temporarily (2.5 months) paused the program during the first wave of the COVID-19 pandemic. This accounts for the 75, 80 and 88% of the total annual transplant activity of the country, respectively (data source: Greek Transplant Organization; 2020).

## 5. Conclusions

It is evident that the annual number of available organs from deceased donors will not resolve the organ shortage. Living donors have thus emerged as an even more important donor category. The superior outcomes of living donor kidney transplantation, the increased willingness of living donors to help their loved ones and the recent advances in LDN have, to some degree, helped to meet the increasing need for kidney allografts. The price we certainly pay is a growing dependency on healthy individuals who undergo a major surgical procedure with no direct therapeutic benefit.

At the transplantation unit in “LAIKO” General Hospital of Athens, the annual number of living donor kidney transplantations increased from 20 to almost 75 within a 5-year period, while the number of deceased donor transplantations remains constantly low. Today, living donation around the world has increasingly become the treatment of choice for patients with ESRD. Transplant centers should demonstrate sufficient volume of surgical procedures and training (especially minimally invasive donor nephrectomy) to ensure a high level of surgical skills and state-of-the art care of the living donor.

In view of the increasing kidney demand, the transplant community should continuously reevaluate the accepted exclusion criteria for donors. In the future, more “complex” living donors will have to be accepted. From our study, it is obvious that even those donors may safely donate with very good results.

## Figures and Tables

**Table 1:** Patient characteristics.

Type of Nephrectomy	Open = 135		Laparoscopic = 117		
	Mean	SD	Mean	SD	*p*-Value
Age (years)	58	0.89	58	0.99	0.77 ^1^
Cr at operation (mg/dL)	0.73	0.01	0.73	0.01	0.97 ^1^
	Median	p25–p75	Median	p25–p75	
eGFR (CKD–EPI) at op (mL/min/1.73 m^2^)	93	86–100	95.5	83.5–101	0.52 ^2^
	*N*	%	*N*	%	
Gender (Male/Female)	39/96	29/71	31/86	26/74	0.39 ^3^
Kidney (Right/Left)	17/118	12.6/87.4	16/101	13.7/86.3	0.79 ^3^
Hypertension (No/Yes)	109/26	80.7/19.3	100/17	85.4/14.6	0.32 ^3^
Past abdominal surgery (No/Yes)	108/24	81.8/18.2	71/46	60.7/39.3	**<0.001 ^3^**
Preemptive Tx (No/Yes)	108/24	81.8/18.2	101/16	86.3/13.7	0.33 ^3^
ABOi–Tx (No/Yes/Specificity)	122/11/0	91.7/8.3/0	89/26/2	76.1/22.2/1.7	**0.001 ^4^**

SD: standard deviation, Cr: creatinine, BMI: body mass index, eGFR: estimated glomerular filtration rate, CKD–EPI: chronic kidney disease epidemiology collaboration equation, op: operation, Tx: transplantation, ABOi–Tx: ABO-incompatible transplantation. ^1^: *t*-test test for independent samples for continuous variables; ^2^: Mann–Whitney U test for independent samples for continuous variables; ^3^: Chi-square test for categorical variables; ^4^: Fischer’s exact test for categorical variables. Statistical significance: *p* < 0.05, mentioned in bold.

**Table 2:** Subgroup analysis by different age and BMI categories.

	*N*	%	*N*	%	*p*-Value
Age (years)					0.19
≤60	71	52.6	71	60.7	
>60	64	47.4	46	39.3	
Age (years)					0.75
≤65	98	72.6	87	74.3	
>65	37	27.4	30	25.7	
BMI (kg/m^2^)					0.008
≤30	119	88.1	88	75.2	
>30	16	11.9	29	24.8	

BMI: body mass index.

**Table 3:** Major outcomes in the two groups.

Type of Nephrectomy	Open = 135		Laparoscopic = 117		
	Mean/SD		Mean/SD		*p*-Value
Cr at discharge (mg/dL)	1.09/0.01		1.15/0.02		**0.04** ^**1**^
	Median	p25–p75	Median	p25–p75	
eGFR at discharge	60	52–71	59	48–65	**0.03** ^**2**^
Duration of surgery (min)	240	230–245	160	160–170	**<0.01** ^**2**^
Warm ischemia time (min)	2	2–2	6	6–8	**<0.01** ^**2**^
Duration of hospitalization (days after surgery)	7	6–8	3	3–4	**<0.01** ^**2**^
Blood loss (mL)	20	10–20	50 mL/1 pt	–	–
	*N*	%	*N*	%	
Transfusion (No/Yes)	130/2	98.5/1.5	–	–	–
Complications (No/Yes)	113/22	83.7/16.3	99/18	84.6/15.4	0.84 ^3^
Complications (Specific)					
Wound infection	4	2.9	–	–	
Wound infection + postop pain	8	5.8	–	–	
Abdominal wall relaxation	9	6.7	–	–	
Wound seroma	–	–	3	2.6	
Chylousascites	–	–	3	2.6	
Other	1	0.7	12	10.4	

Cr: creatinine, eGFR: estimated glomerular filtration rate, postop: postoperative. ^1^: *t*-test test for independent samples for continuous variables; ^2^: Mann–Whitney U test for independent samples for continuous variables; ^3^: Chi-square test for categorical variables. Statistical significance: *p* < 0.05, mentioned in bold.

**Table 4:** Comparison of SF-36 scores between two groups.

Questionnaire Domains	Laparoscopic				Controls				
	*N*	Median	p25	p75	*N*	Median	p25	p75	*p* Value
SF-1	119	100	100	100	122	90	85	95	<0.001
SF-2	119	100	100	100	122	100	100	100	<0.001
SF-3	118	100	100	100	120	100	82	100	<0.001
SF-4	118	100	82	100	122	82	72	92	<0.001
SF-5	119	100	100	100	122	60	50	70	<0.001
SF-6	118	100	100	100	121	100	87.5	100	<0.001
SF-7	119	100	100	100	122	100	100	100	<0.001
SF-8	119	100	100	100	122	56	52	64	<0.001
SF-PCS	117	57.87	55.68	57.87	119	57.07	53.18	59.76	0.89
SF-MCS	117	62.14	62.14	62.16	119	45.22	41.60	49.70	<0.001

The 8 different domains of the SF-36 questionnaire and the 2 summary scores are presented. Each one is expressed as median with 1st (p25) and 3rd (p75) quartiles. To investigate the differences between groups, the Mann–Whitney U test for independent samples for continuous variables was applied. SF-36 is a multi-item scale measuring 8 health domains: physical functioning (PF), physical role (RP), bodily pain (BP), general health (GH), vitality (VT), social functioning (SF), emotional role (RE) and mental health (MH). Two summary scores are also included: mental component summary score (MCS), physical component summary score (PCS).

## Data Availability

Data are not available publicly due to ethical restrictions.
